# A Prophylactic Effect of Quinine against Erysipelas

**Published:** 1852-09

**Authors:** A. J. Wedderburn

**Affiliations:** Professor of Anatomy in University of Louisiana


From the New Orleans Monthly Medical Register.

*The Prophylactic Effect of Quinine against Erysipelas.* A paper read before the last session of the Louisiana State Medical Society, by A. J. WEDDERBURN, M. D., Professor of Anatomy in University of Louisiana.

THE object of this paper is to direct the attention of the medical profession towards the beneficial results obtained from the use of the sulphate of quinine, not only as a powerful remedy in the treatment of erysipelas, but to its great virtues as a prophylactic against this universal scourge of the surgical wards of the hospitals of our own country and Europe. I will, therefore, first call your attention to a few cases reported some years since in the New Orleans Medical Journal, concerning the use of this remedy in the treatment of ulcers, and other surgical affections; and I will then be enabled to point out how its beneficial results in the treatment of hospital gangrene and erysipelas led to the investigations exhibiting its prophylactic effects.

The following was published in the New Orleans *Medical and Surgical Journal*, January, 1846:

"On taking charge of three surgical wards in the Charity Hospital about the 1st of November last, I found a number of cases of ulcer of an indolent character, some of which had been in the hospital many months without improvement, although the treatment pursued had been varied from time to time, according to the conditions presented by the ulcers.

From the powerful remedial effects, produced by the sulphate of quinine, in the various diseases in which it has been applied so largely of late, by many physicians of the South, and from the recollection of its action in arresting the ulcerative process in a very remarkable manner in an ulcer of the leg from a compound comminuted fracture of the same, in a case occurring in my practice several years since, I was induced to believe good effects might be obtained from its topical application in the above-mentioned ulcers, from a conjecture that the quinine in substance would act as a local stimulant, and by its absorption as a general tonic. I therefore had it applied in twelve or fifteen cases, and found on the next day all, with one or two exceptions, had undergone a change for the better, and that in a few days the surfaces of indolent ulcers had assumed a healthy appearance. I have been using this remedy in several cases of extensive ulcer of the leg, since I first commenced its use, and in every case the improvement has been rapid, and in no case have I had reason to substitute any other remedy. The following are a few of the most striking cases :

CASE I.—A. K., aged 28 years, entered the hospital on the 31st of August, with a phagedenic ulcer of two and a half months' standing, with caries of the tibia. Before I took charge of the ward a large portion of the bone had exfoliated. When I first saw the case the ulcer extended from the external malleolus to within four inches of the patella, with an extent in breadth of about four inches, and at the upper and middle part, one inch in depth, and half an inch at the lower. The lower part of the ulcer presented several dark spots with sinuous openings, discharging a sanious fluid—while the upper portion presented the general characters of an indolent ulcer. The entire surface was ordered to be covered with equal parts of quinine and flour, and in a few days the character of the ulcer was entirely changed, presenting a healthy granulating surface throughout the whole extent, and secreting healthy pus. At the present time it is reduced in length to about five inches, in breadth two, and the greatest depth about a half an inch. Since the commencement of the treatment, nothing has been used but the sulphate of quinine, except on two occasions there appeared to be a too rapid growth of granulations, and the quinine in substance was withheld, and a mixture of tannin grs. ij. and the sulph. quinine grs. v. to the ounce of water, was applied during one or two days.

CASE II.—D. D., aged 35 years, entered the hospital on the 23rd November, with an indolent ulcer immediately above the external malleolus, circular in form, and near three inches in diameter, more than half an inch in depth, and at the posterior part a deep sinus penetrating beneath the tendo Achilles. After using as a topical application to this ulcer, Labaraque's chloride of soda five or six days without the slightest improvement, the sulphate of quinine was resorted to, and the character of the ulcer entirely changed in twenty-four hours. The same treatment has been continued up to the present time; the diameter of the ulcer is much diminished, the granulations are on a level with the surface, and the new skin is forming rapidly.

CASE III.—C. S., aged 38 years, entered the hospital on the 8th December, with a sloughing phagedenic ulcer of the penis, from primary syphilis. About two-thirds of the skin had separated from the dorsum of the penis, exposing the corpora cavernosa. The separation at the under part had not taken place at this time between the living and the dead parts. The discharge was sanious, and very offensive. Ordered the parts to be washed with the chloride of soda, and apply an anodyne poultice. Second day, no improvement—ulceration progressing rapidly upon the corona glandis; discharge continues to be offensive—pain great, with great constitutional disturbance. Ordered the whole penis to be enveloped in the sulphate of quinine and flour in equal parts. Third day—ulcerative process entirely arrested, the whole surface, except the sphacelus on the dorsum, covered with granulations, and the secretions healthy, little or no pain, and no constitutional derangement. The ulcer is now healing rapidly.

CASE IV.—The operation for phymosis by circumcision, was performed on one of the inmates of the hospital about ten days ago. The day after the operation, the penis was in an œdematous and inflamed condition—the inflammation of the erysipelatous kind; a solution of five grs. of the acetate of lead to the ounce, was continually applied during the day. The next day solutions of tannin and the sulphate of quinine were applied, with good results; on the third day the sulphate of quinine and flour was applied to the cut surface, and continued for several days, until the inflammation had nearly subsided, when an ointment of the acetate of lead was ordered, with a view to favor the healing process. On dressing the ulcer twenty-four hours afterwards, the penis was found again very much enlarged, and highly inflamed, with very great pain. The sulphate of quinine was again applied, in substance to the part, and over this a poultice containing a solution of the sulphate of quinine and tannin. The improvement was so great the next day that the tannin was withheld, and the quinine applied to the surface with a light elm poultice. This treatment has now been continued for three days, and there is every prospect of a rapid recovery."

Several cases of phlegmonous erysipelas; two of the leg, one of the thigh, and two of the fore arm, are mentioned in this report, but amongst these is found a description of one in which the disease was so formidable as to have produced the almost entire destruction of the sub-cutaneous cellular tissue from the ancle to within a short distance of the knee-joint. An opening was made over the spine of the tibia, about two inches in length, from which there was a large discharge of pus. The discharge continuing for several days to be very profuse, a counter opening was made at the back of the leg, and in a few days the injury had been sufficiently repaired to enable him to leave the hospital; at times the constitutional symptoms were very alarming, but yielded readily to large doses of the sulphate of quinine, combined with opium. A solution of quinine about eight grs. to the ounce, was the chief topical treatment resorted to in this case.

In the September number of the Journal, for 1846, we find the following:

"Since the report of the January number of this Journal, concerning the treatment of indolent ulcers in my wards at the Charity Hospital with the sulphate of quinine, I have continued its topical application, not only to indolent ulcers, but have used it with marked success in other varieties.

"I have noticed the following facts:—that when applied to the surface of an inflamed ulcer, the redness surrounding the part has been subdued in less than twenty-four hours, and the same holds good concerning the pain in an irritable ulcer, whilst almost invariably in the same space of time the surface is made to present an healthy, granulating appearance. When this remedy is applied to a small indolent ulcer, its effect is not so prompt as when applied to a large ulcer of the same variety. Now, this would lead us to suppose that the remedy is absorbed; but from experiments, I have ascertained that the statements made by M. Martin Solon in the *Bulletin de Therapeutique*, and republished in the March number of this Journal, are entirely correct, viz.: that the sulphate of quinine when applied to the surface, is not absorbed; *i. e.*, if its not being detected in the urine is evidence of this fact. I have always been able to detect it when given internally in small quantities; when I have failed at the same time, in testing the urine of those when it had been applied to the surface. In a case of phlegmonous erysipelas of the thigh of a woman in the Charity Hospital, extending over a surface of eight or ten inches in length, and occupying its entire breadth on its external part, with an opening in the centre about an inch in diameter; under the inflamed surface the cellular tissue so completely destroyed, that a probe could be passed freely in any direction. Into this large sinus, an ounce of water holding in solution ten grs. of quinine was placed, three times during the day, making in all 30 grs. The woman was placed in such a position that the escape of the solution was entirely prevented. The next day the pain and redness of the surface had nearly disappeared; and it was clear that the three ounces of the solution (*thirty grs. of quinine*) had permeated the tissues. The urine voided before the administration of this remedy, and that passed during its administration, was tested with all the tests for quinine, and the effects produced upon both were the same, no precipitate being afforded in either case. This case recovered in a very short time, no other remedy having been used during the treatment."

Passing here over a number of cases of ulcer, successfully treated with the sulphate of quinine, I will only mention the following case:

A. R., was admitted into the hospital, with an extensive syphilitic ulcer extending from the upper part of the sacrum to the anus, exposing a large portion of the ligaments covering the sacrum—this case had been under treatment for more than ten days—various applications had been made without the slightest effect, when I determined to resort to the topical application of the sulphate of quinine. I ordered the part to be washed with a solution of tannin eight grains to the ounce of water, and lint to be constantly applied saturated with a solution of quinine, ten grs. to the ounce. The constitutional symptoms under which she was suffering, with the excessive pain of the part, were much relieved in about twenty-four hours, and in three days the entire surface was covered with healthy granulations. It is now about twenty days since the quinine was first applied—the ulcer has improved daily since its first application, and will be entirely cured in a few days, as the granulations are now even with the surface of the skin.

Before calling your attention particularly to the prophylactic effect of quinine in erysipelas, I desire to make a few remarks concerning its remedial effects, from further and more recent observations in the treatment of ulcers, which are intended to confirm fully the statements already made. It is not my intention to occupy the time of the society by reading a long list of cases which could be given, but will content myself with mentioning that this remedy has not only proved useful in the treatment of such ulcers as have been already recorded, but that in the treatment of ulcers of the cornea I have invariably found it much more prompt in giving relief, than the nitrate of silver—the solution used in these cases is very strong, viz. : twelve grs. of quinine to two drams of water, with twelve or fifteen drops of elixir vitriol. The number for November, 1846, of the New Orleans Medical Journal contains a correspondence between Dr. Fearn and myself, in which he mentions the good results obtained from the topical use of a solution of quinine in the treatment of conjunctivitis and urethritis.

Coming now to the consideration of the prophylactic effect of our remedy, I have simply to state, that from the powerful remedial effects witnessed in its topical application to cut surfaces affected with erysipelas, and from the fact that this form of inflammation almost invariably followed the use of the knife in the wards of the Charity Hospital, I was induced as far back as the winter of 1846–47, to dress, after all operations, with a solution of quinine, hoping, through its agency, to ward off this formidable disease. Since the time mentioned, I have constantly used it immediately after operation, (whenever the disease has been in the hospital,) and in no single instance have I known it occur after the application.

During the winter of 1849–50, there were a much larger number of operations in the hospital than were ever made before in the same space of time, which gave me an ample opportunity of obtaining further evidence concerning the subject in question. One of the most important cases in which this remedy was used, was a case in which I removed the entire half of the lower jaw by disarticulation. After the operation, before bringing the edges of the wound together, and securing them with the twisted suture, the surface was washed with a solution of quinine, containing four grs.

to the ounce of water, and after application of the sutures, lint, saturated with the same solution, was applied to the part and continued during the progress of the case, which resulted in healing by the first intention. This case was in a ward where there were several bad cases of erysipelas, and it was prevailing violently throughout the hospital. I could continue this subject by the recital of a vast number of cases showing clearly the prophylactic effect of quinine, but am not willing to occupy more of your time, and will therefore conclude by requesting those who doubt these facts, to endeavor to confirm their doubts by trying the remedy.

